# Assembly of 500,000 inter-specific catfish expressed sequence tags and large scale gene-associated marker development for whole genome association studies

**DOI:** 10.1186/gb-2010-11-1-r8

**Published:** 2010-01-22

**Authors:** Shaolin Wang, Eric Peatman, Jason Abernathy, Geoff Waldbieser, Erika Lindquist, Paul Richardson, Susan Lucas, Mei Wang, Ping Li, Jyothi Thimmapuram, Lei Liu, Deepika Vullaganti, Huseyin Kucuktas, Christopher Murdock, Brian C Small, Melanie Wilson, Hong Liu, Yanliang Jiang, Yoona Lee, Fei Chen, Jianguo Lu, Wenqi Wang, Peng Xu, Benjaporn Somridhivej, Puttharat Baoprasertkul, Jonas Quilang, Zhenxia Sha, Baolong Bao, Yaping Wang, Qun Wang, Tomokazu Takano, Samiran Nandi, Shikai Liu, Lilian Wong, Ludmilla Kaltenboeck, Sylvie Quiniou, Eva Bengten, Norman Miller, John Trant, Daniel Rokhsar, Zhanjiang Liu, the Catfish Genome Consortium

**Affiliations:** 1 The Fish Molecular Genetics and Biotechnology Laboratory, Department of Fisheries and Allied Aquacultures and Program of Cell and Molecular Biosciences, Aquatic Genomics Unit, 203 Swingle Hall, Auburn University, Auburn, AL 36849, USA; 2 USDA, ARS, Catfish Genetics Research Unit, 141 Experiment Station Road, Stoneville, Mississippi 38776, USA; 3 DOE Joint Genome Institute, Genomic Technologies Department, 2800 Mitchell Drive Bldg 400-462, Walnut Creek, CA 94598, USA; 4 The WM Keck Center for Comparative and Functional Genomics, University of Illinois at Urbana-Champaign, Urbana, IL 61801, USA; 5 Department of Microbiology, University of Mississippi Medical Center, 2500 North State Street, Jackson, MS 39216, USA; 6 Center of Marine Biotechnology, University of Maryland Biotechnology Institute, 701 East Pratt Street, Baltimore, MD 21202, USA; 7 Department of Molecular and Cell Biology, University of California, Berkeley, 142 Life Sciences Addition #3200, Berkeley, CA 94720, USA

## Abstract

**Background:**

Through the Community Sequencing Program, a catfish EST sequencing project was carried out through a collaboration between the catfish research community and the Department of Energy's Joint Genome Institute. Prior to this project, only a limited EST resource from catfish was available for the purpose of SNP identification.

**Results:**

A total of 438,321 quality ESTs were generated from 8 channel catfish (*Ictalurus punctatus*) and 4 blue catfish (*Ictalurus furcatus*) libraries, bringing the number of catfish ESTs to nearly 500,000. Assembly of all catfish ESTs resulted in 45,306 contigs and 66,272 singletons. Over 35% of the unique sequences had significant similarities to known genes, allowing the identification of 14,776 unique genes in catfish. Over 300,000 putative SNPs have been identified, of which approximately 48,000 are high-quality SNPs identified from contigs with at least four sequences and the minor allele presence of at least two sequences in the contig. The EST resource should be valuable for identification of microsatellites, genome annotation, large-scale expression analysis, and comparative genome analysis.

**Conclusions:**

This project generated a large EST resource for catfish that captured the majority of the catfish transcriptome. The parallel analysis of ESTs from two closely related Ictalurid catfishes should also provide powerful means for the evaluation of ancient and recent gene duplications, and for the development of high-density microarrays in catfish. The inter- and intra-specific SNPs identified from all catfish EST dataset assembly will greatly benefit the catfish introgression breeding program and whole genome association studies.

## Abstract 2

Twelve cDNA libraries from two species of catfish have been sequenced, resulting in the generation of nearly 500,000 ESTs.

## Background

Catfish is the major aquaculture species in the United States, accounting for over 60% of all US aquaculture production. While channel catfish (*Ictalurus punctatus*) accounts for the majority of commercial aquaculture production, the closely related blue catfish (*Ictalurus furcatus*) possesses several economically important traits that led to the production of an inter-specific hybrid (channel catfish female × blue catfish male) available for commercial use [1]. This specific hybrid shows strong heterosis and superior performance traits in disease resistance, growth rate, feed conversion efficiency, processing yields, and seinability. Channel catfish is also an important model species for the study of comparative immunology, reproductive physiology, and toxicology. The channel catfish immune system is among the best characterized of any fish species, with decades of research leading to identification and characterization of catfish immune genes [2,3], establishment of clonal functionally distinct lymphocyte cell lines [4], characterization of much of the machinery of catfish innate [5,6] and adaptive immunity and production of panels of specific monoclonal antibodies for detection of catfish immunocytes [7-9].

Genome research requires the development of a number of resources that facilitate both structural and functional analysis of the genome. Many of the required resources have been developed in catfish, including a large number of polymorphic markers [10,11], linkage maps [12-14], bacterial artificial chromosome (BAC) libraries [15,16], physical maps [17,18], and BAC end sequences (BES) [19,20]. However, expressed sequence tag (EST) resources were low from catfish [21-26], hindering both functional and comparative genome analysis in catfish. Large numbers of ESTs have been produced for most model species as well as a number of agriculturally important species [27-32], including cattle (1.5 million), swine (1.4 million), chicken (600,000), Atlantic salmon (471,000), and rainbow trout (281,000). The availability of such EST resources has allowed efficient gene discovery and gene identification in these species, and rapid progress has been made through comparative genome analysis in understanding structural, organizational, and functional properties of the genomes of these species.

A whole genome sequence is not available for most aquaculture species. In the absence of the whole genome sequence of catfish, we initiated this large-scale EST project to provide transcriptomic resources in channel catfish and blue catfish. These ESTs will serve as resources for gene discovery and gene identification, supply the framework for high-density microarray platforms, provide a foundation for the analysis of full-length cDNAs, and assist in the identification of genetic markers such as microsatellites and single nucleotide polymorphisms (SNPs). In this study, we have taken a unique inter-specific approach. The inter-specific approach will help develop markers that are inter-specific and species specific. These resources will also be of great use for comparative genome analysis. The inter-specific EST approach to produce parallel EST resources from two closely related Ictalurid species will allow the resolution of some of the most difficult issues in teleost genome research, such as paralog confusions involving duplicated genomes [33-35]. Here we report the generation and analysis of nearly 500,000 ESTs from catfish, including 354,377 ESTs from channel catfish and 139,475 ESTs from blue catfish.

## Results

### cDNA libraries and EST sequencing

**Figure 1:**
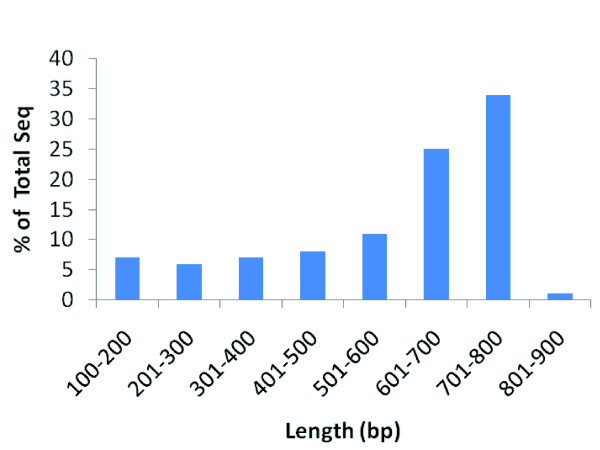
**Length distribution of Joint Genome Institute EST sequences**.

Twelve cDNA libraries were constructed from various tissues, organs, and cell lines, including four blue catfish libraries and eight channel catfish libraries (Table 1). More than 600,000 sequencing reactions were attempted to sequence a total of 307,296 cDNA clones from both ends. A total of 438,321 ESTs were generated from this project, of which 128,711 sequences were from blue catfish and 309,610 were from channel catfish (Table 1). Of these EST sequences, 219,831 were sequenced from the 5' end of the transcripts, and 218,490 were sequenced from the 3' end. A total of 194,136 clones had paired reads from both 5' and 3' ends of the same transcripts. The lengths of the ESTs range from 100 to 877 bp, with an average length of 576 bp and a median length of 655 bp (Figure 1). Addition of these sequences to the 10,764 blue catfish ESTs and 44,767 channel catfish ESTs in GenBank before the start of this project increased the number of catfish ESTs to almost a half million sequences (139,475 blue catfish ESTs and 354,377 channel catfish ESTs; Table 1). The ESTs from blue catfish and channel catfish have been deposited in GenBank under accession numbers [GenBank:FC996013-FC999999, FD000001-FD380635 and GH640296-GH693994].

**Table 1:** cDNA library information and sequencing summary

Library	Species	Nature of library	Organ, tissue, or cell line	Total sequences
CBFH	Blue catfish	Normalized	Stomach, muscle, olfactory tissue and trunk kidney	37,314
CBZC	Blue catfish	Normalized	Stomach, muscle, olfactory tissue and trunk kidney	30,902
CBNH	Blue catfish	Normalized	Head kidney, gill, intestine, spleen, skin and liver	9,323
CBZF	Blue catfish	Normalized	Head kidney, gill, intestine, spleen, skin and liver	51,172
Subtotal				128,711
				
CBCZ	Channel catfish	Non-normalized	Mixed leukocytes of parallel blood leukocytes	16,168
CBFA	Channel catfish	Normalized	Catfish whole fry library	63,602
CBNG	Channel catfish	Normalized	Kidney, gill, intestine, spleen, skin and liver	2,982
CBZB	Channel catfish	Normalized	Kidney, gill, intestine, spleen, skin and liver	57,772
CBNI	Channel catfish	Normalized	Stomach, muscle, olfactory tissue and trunk kidney	17,023
CBZA	Channel catfish	Normalized	Stomach, muscle, olfactory tissue and trunk kidney	61,320
CBPN	Channel catfish	Subtracted	Liver, pituitary, ovary and testis	62,058
CBPO	Channel catfish	Normalized	Peripheral blood leukocytes stimulated with LPS	28,685
Subtotal				309,610
				
NCBI	Blue catfish			10,764
NCBI	Channel catfish			44,767
				
Total				493,852

Library names were designated by the Joint Genome Institute. LPS, lipopolysaccharide.

### EST assembly

All existing catfish EST sequences were used to produce three assemblies: blue catfish ESTs; channel catfish ESTs; and blue catfish and channel catfish ESTs for inter-specific analysis. Assembly of 139,475 blue catfish ESTs resulted in 54,815 unique sequences (22,009 contigs and 32,806 singletons) whereas assembly of 354,377 channel catfish ESTs resulted in 70,717 unique EST sequences (28,941 contigs and 41,776 singletons) (Table 2). Details of the catfish EST assembly are available online [36].

**Table 2:** EST assembly statistics

	Blue catfish	Channel catfish	All catfish
	139,475	354,377	493,852
Short and simple sequences removed	2,735	6,230	8,965
Sequences for assembly	136,740	348,147	484,887
Contigs	22,009	28,941	45,306
Singletons	32,806	41,776	66,272
Average number of sequences per contig	4.72	10.6	9.2
Total unique sequences	54,815	70,717	111,578

**Figure 2:**
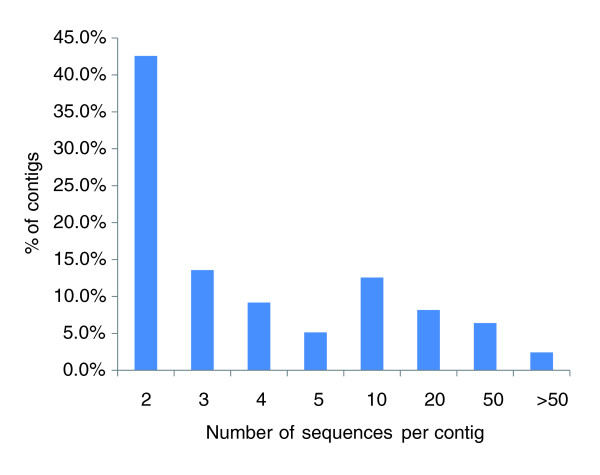
**Distribution of contig sizes**.

In order to identify inter-specific SNPs, we also conducted the assembly of all available 493,852 ESTs from blue catfish and channel catfish. This assembly allowed the formation of 45,306 contigs (66,272 singletons), from which potential inter-specific SNPs could be identified. The number of inter-specific contigs was significantly larger than that from either species, potentially due to the formation of new contigs of related transcripts that were singletons in either species (see Discussion). A majority of contigs contained only two (43%) or three (13%) sequences (Figure 2), and average contig depth was nine sequences. With the ESTs being sequenced mostly from normalized libraries, the vast majority of contigs had 50 or fewer sequences. However, some extremely large contigs were found. The largest contig, containing 7,208 sequences, putatively identified as apolipoprotein, was repeatedly sequenced from all libraries, and was prevalent in the pre-existing non-normalized libraries in GenBank. As previously reported [37], contig depth is one of the two most important factors affecting EST-derived SNP qualities. Therefore, the information on contig depth is highly useful.

**Figure 3:**
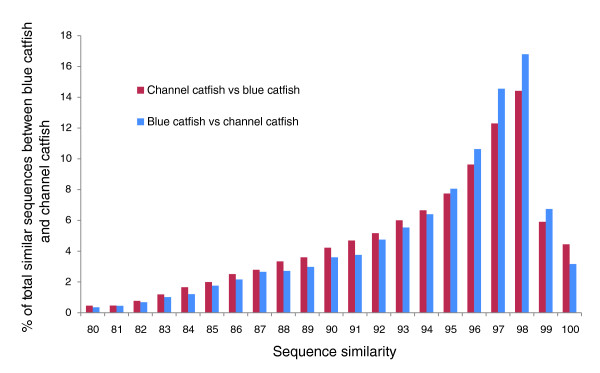
**Distribution of sequence similarity between blue catfish and channel catfish sequences**.

To assess the similarity between blue catfish and channel catfish sequences, the consensus sequences from each species were compared to each other using BLASTN at a stringency of 1E-10. Of blue catfish and channel catfish sequences with a minimal 200-bp matching region, sequence similarity ranged from 77% to 100%, with an average similarity of 95%. Over 50% of blue catfish and channel catfish homologous sequences have similarity levels over 95% (Figure 3).

### Gene identification and annotation

**Figure 4:**
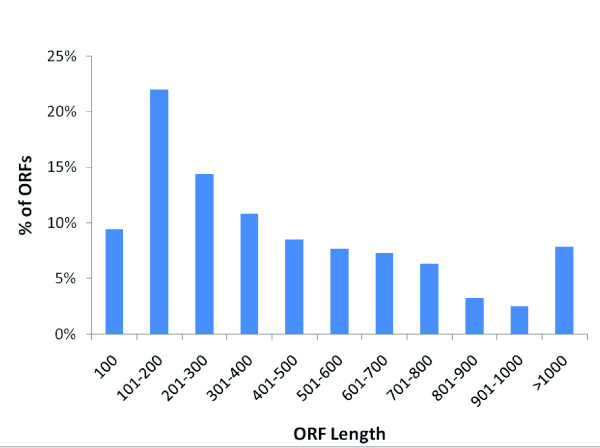
**Open reading frame (ORF) length distribution from unique sequences of the all catfish assembly**.

**Figure 5:**
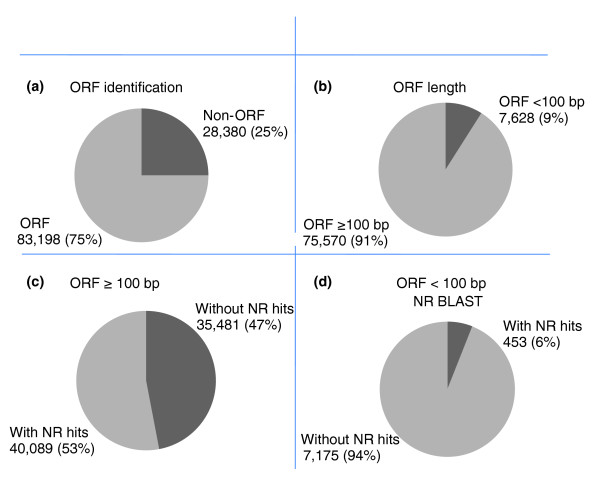
**Analysis of open reading frames (ORFs)**. **(a) **Percentage of ORFs among unique sequences from the all catfish EST assembly; **(b) **Percentage of ORF greater than 100 bp among unique sequences from the all catfish EST assembly; **(c) **Percentage of ORFs equal to or greater than 100 bp with significant BLASTX hits; **(d) **Percentage of ORFs smaller than 100 bp with significant BLASTX hits

Putative gene identification was conducted either by *ab initio *identification of open reading frames (ORFs) or by BLASTX similarity search of public protein databases. Of the 111,578 total unique catfish sequences (total catfish EST assembly), ORFs were detected from 83,198 (75%) unique sequences, with an average ORF length of 450 bp (minimum = 51 bp, maximum = 14,674 bp; Figure 4), and the remaining 28,380 sequences (25%) contained no ORFs (Figure 5a). These ORF-less ESTs were likely ESTs sequenced within the untranslated regions (UTRs) or within intron-retaining cDNAs.

There was a positive correlation between the length of ORF and BLASTX match. Of the identified ORFs, 91% had a length of more than 100 bp. Within these ORFs, 53% had significant (1E-10) BLASTX matches (Figures 5b, c). However, only 6% of the ORFs with less than 100 bp had significant BLASTX matches (Figure 5d).

A total of 41,311 (37%) unique sequences had significant BLASTX matches within the *nr *database, and 34,860 (31%) had significant BLASTX matches within the Uniprot database (Table 3). Over 98% of unique sequences with significant matches were identified with ORFs, which indicated the reliability of ORF searching. After examination of putative protein identities from the BLASTX searches, homologous sequences were identified from the catfish ESTs. Of the 41,311 sequences with BLASTX hits, 22,642 (approximately 55%) and 17,948 (approximately 43%) unique proteins were identified through searches against the *nr *and the Uniprot protein databases after removing the redundant protein hits, respectively.

**Table 3:** Summary of BLASTX search analysis of catfish ESTs

Database	Catfish hits*	Unique protein	% of total unique proteins	Unique gene
NR	41,311	22,642		
Uniprot	34,860	17,948		
Refseq/Ensembl				
Zebrafish	39,546	14,988	54% of 27,996	12,470
Medaka	36,641	13,588	56% of 24,461	12,920
*Tetraodon*	34,418	13,132	57% of 23,118	10,322
Human	33,847	12,621	33% of 38,342	9,668
Mouse	33,594	12,267	35% of 35,236	11,518
Chicken	31,646	11,059	50% of 22,194	8,717
Cumulative unique (E^-10^)^†^	42,668	16,439		14,776

*Number of significant (E^-10^) alignments using all catfish unique sequences as queries to search the listed databases. ^†^Cumulative unique totals were derived from the sum of unique gene/protein identities across all listed species.

### Assessment of the sequenced catfish transcriptome

In order to assess the level to which the catfish transcriptome has been captured, the unique catfish sequences (111,578) were also searched against the NCBI Refseq and Ensembl databases. A number of significant hits were identified within zebrafish, medaka, *Tetraodon*, human, mouse, and chicken reference protein databases (Table 3). After removal of the redundant protein hits, 14,988 - 11,059 unique reference proteins were identified within zebrafish, medaka, *Tetraodon*, human, mouse, and chicken databases respectively (Table 3). The unique catfish sequences had hits to 54% to 57% of the unique proteins of zebrafish, medaka, and green-spotted pufferfish. To allow comparison of catfish unique protein coverage with that expected between species with complete genome sequences, all *Tetraodon *Ensembl proteins were searched against the medaka Ensembl protein database. A total of 22,150 *Tetraodon *proteins have significant hits to 15,054 (61% of total unique) medaka Ensembl proteins with a cutoff E-value of 1E-10. Similarly, zebrafish Refseq proteins were searched against the human Refseq protein database. In this case, 24,971 zebrafish proteins have significant hits to 13,789 (36% of total unique) human proteins with a cutoff E-value of 1E-10. Taken together, these numbers provide strong evidence that this project has captured a large majority of the catfish transcriptome.

**Figure 6:**
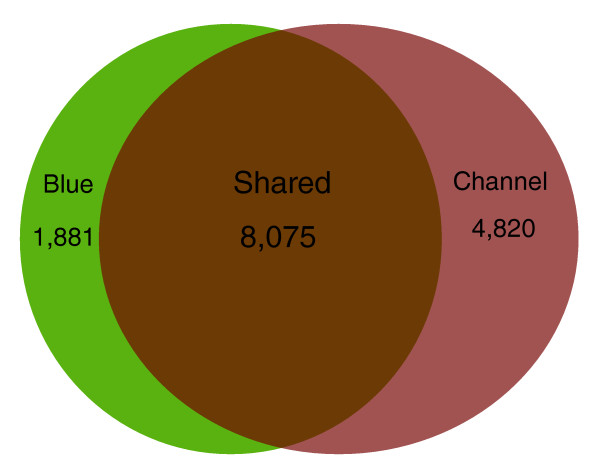
**Comparison of shared and unique gene identities of channel catfish and blue catfish from a total of 14,776 unique genes**.

A total of 14,776 cumulative unique genes were identified from catfish based on BLASTX searches against the Refseq/Ensembl database (Table 3), including 8,075 genes identified from both blue catfish and channel catfish, 1,881 unique genes from blue catfish and 4,820 unique genes from channel catfish (Figure 6). As expected based on sequencing coverage depths, significantly more unique genes were identified from channel catfish than blue catfish.

**Figure 7:**
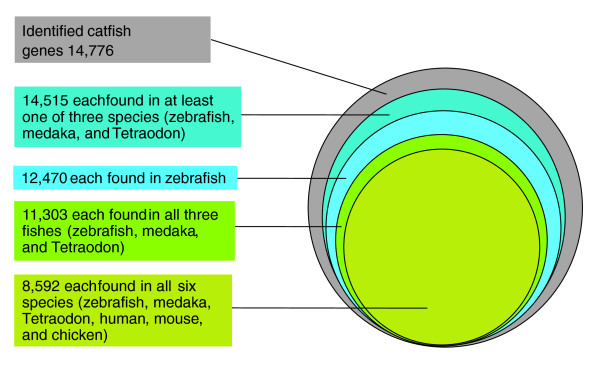
**Conservation of catfish gene identities with other species**. Number of catfish homologous genes identified from other species using BLASTX searches.

To assess the evolutionary conservation of the identified unique genes, the number of hits to unique genes in each species of zebrafish, medaka, *Tetraodon*, human, mouse, and chicken were compared. A total of 8,592 (58% of total number of unique catfish genes) putative known unique genes were found in all six species: 11,303 (76%) were found in all three fish species; and 14,515 (98%) were found in at least one of the three fish species (Figure 7), indicating a high level of conservation of gene content among catfish and other teleost fish species.

### Prediction of full-length cDNAs

The catfish EST sequences provide a platform for the identification and characterization of full-length cDNA clones without having to use expensive and labor-intensive primer walking sequencing. In the context of this work, full-length cDNA inserts were defined as a cDNA from a single clone with the start codon and poly (A) tail contained within the clone. A total of 10,037 channel catfish and 7,382 blue catfish putative full-length cDNAs were identified from the assembly with a cutoff E-value of 1E-5. A well characterized full-length cDNA set from catfish will be crucial in ongoing studies of teleost gene duplication and gene family structure, as well as aiding in annotation of the catfish whole genome sequence. Current efforts are focused, therefore, on characterization and re-sequencing of these full-length cDNAs.

### Microsatellite and SNP marker identification

A total of 20,757 microsatellites were initially identified from 15,082 unique sequences, including di-, tri-, tetra-, penta- and hexa-nucleotide repeats (Table 4). After removing the microsatellites without enough flanking sequence for primer design, 13,375 unique sequences with microsatellites had sufficient flanking sequences (50 bp) on both sides of the microsatellites to design primers for genotyping. Our previous research indicated that over 72% of EST-derived microsatellites were polymorphic in one resource family [12], suggesting the potential utility of these microsatellites.

**Table 4:** Summary of microsatellite marker identification from catfish ESTs

Total number of unique sequences	111,578
Microsatellites identified	20,757
Di-nucleotide repeats	12,367
Tri-nucleotide repeats	5,506
Tetra-nucleotide repeats	2,664
Penta-nucleotide repeats	182
Hexa-nucleotide repeats	38
Number of unique sequences containing microsatellites	15,082
Number of unique sequences containing microsatellites with sufficient flanking sequences for PCR primer design	13,375

A total of 48,702 putative SNPs and 14,803 putative insertions/deletions (indels) were identified from the blue catfish EST dataset assembly; 102,252 putative SNPs and 41,660 putative indels were identified from channel catfish EST dataset assembly (Table 5). These putative SNPs indicated an SNP rate of 3.2 SNPs per kilobase of transcribed sequences in blue catfish, and 4.1 SNPs per kilobase of transcribed sequences in channel catfish. Obviously, such SNP rates were calculated from the total consensus sequence length and, therefore, the deeper the EST sequencing was, the greater the possibility for the identification of a SNP within the consensus sequences.

**Table 5:** Summary of SNP identification from the catfish ESTs

	Number of SNPs		
	
SNP	Blue catfish	Channel catfish	All catfish
Putative			
Transitions	29,305	61,184	172,746
Transversions	19,397	41,068	130,254
Total SNPs	48,702	102,252	303,000
Indels	14,803	41,660	100,636
SNP rate (kb)	3.2	4.1	7.7
			
Filtered putative			
Transitions	2,886	11,012	32,235
Transversions	1,005	4,815	16,359
Total SNPs	3,891	15,827	48,594
Indels	1,070	6,707	19,398
Filtered/Non-filtered rate	7.8%	15.7%	16.2%
SNP rate* (kb)	0.25	0.64	1.6

*SNP rate was calculated by dividing the total number of SNPs excluding indels by the total length (bp) of the consensus sequences of the contigs.

Over 303,000 putative SNPs and 100,000 putative indels were identified from the all catfish EST assembly results (Table 5). EST-derived SNPs are often prone to sequencing errors. Therefore, the putative SNPs were subjected to filtering using only those with contig sizes of at least four sequences and the minor allele presence of at least two sequences in the contigs, and indels were not used for further analysis [37]. These parameters were previously shown to select markers with 70% success rate for genotyping. After filtering, 3,891 and 15,827 SNPs were identified from the blue catfish and channel catfish EST dataset assemblies, respectively. A subset of 48,594 filtered SNPs were obtained from the all catfish EST assembly; these SNPs included 32,235 transitions and 16,359 transversions (Table 5). The filtered SNP frequency in the transcribed sequences was 0.25 SNP in blue catfish, 0.64 SNP in channel catfish, and 1.6 SNP in the all catfish assembly per kilobase. A total of 19,398 filtered insertions and deletions (Indels) were discovered, that is, 0.64 indels per kilobase of the transcribed sequences. Of the 48,594 SNPs, over 90% were identified from the contigs containing 5 or more sequences (Table 6).

**Table 6:** Quality assessment of the filtered putative SNPs identified from the catfish ESTs based on the number of sequences per contig and the sequence frequencies of the minor alleles

Number of sequences in the contig	Number of contigs with SNPs	Number of SNPs	SNP rate (per kb)
2 (1:1)	16,567	96,565	5.2
3 (2:1)	8,374	86,686	10.8
4 (3:1)	5,136	71,155	13.0
Subtotal	30,077	254,406	8.0
			
4 (2:2)	1,528	5,008	0.9
5-6 (2)	3,099	13,725	2.0
7-8 (3)	805	2,659	0.7
9-12 (4)	730	2,376	0.5
13-20 (5)	629	2,307	0.6
21-30 (5)	628	2,864	1.3
31-50 (6)	730	5,052	3.0
51-100 (6)	542	6,379	6.0
101-500 (6)	316	6,580	13.4
>500	31	1,644	15.0
Subtotal	9,038	48,594	1.6
			
Total	39,115	303,000	7.7

**Figure 8:**
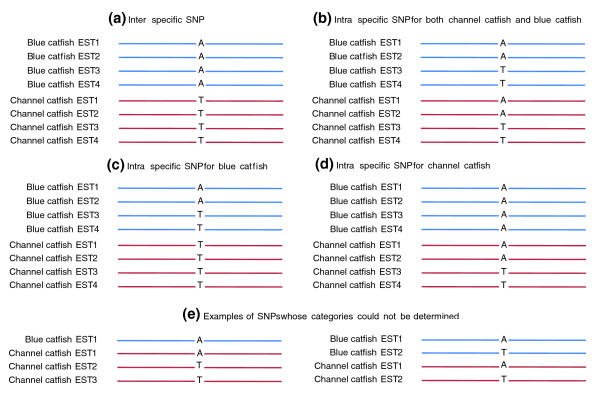
**Categorization of four different types of SNPs from the all catfish EST assembly and examples of SNPs whose categories could not be determined**. **(a-d) **Types of SNPs from the all catfish EST assembly that can be identified from the all catfish EST assembly. **(e) **Examples of SNPs whose categories could not be determined because the minor allele sequence from a given species is fewer than two.

The assessment of the rates of inter-specific SNPs and intra-specific SNPs may have practical applications. We therefore assessed these SNP rates using the EST data. First, SNPs were identified from contigs containing at least four sequences with at least two sequences from either channel catfish or blue catfish in the all catfish EST assembly. Inter-specific SNPs were defined as those that have sequence variations between blue catfish and channel catfish, but no sequence variations within blue catfish or within channel catfish; similarly, SNPs were identified within blue catfish but not within channel catfish or vice versa; and SNPs were identified within both channel catfish and blue catfish at the same SNP positions (Figure 8). Of the 48,594 filtered SNPs, 42,080 were identified from contigs comprising both channel catfish and blue catfish ESTs, and 6,514 were identified from contigs composed of ESTs from either channel catfish or blue catfish, including 5,396 from channel catfish contigs and 1,118 were identified from blue catfish contigs. Of the contigs containing ESTs from both blue catfish and channel catfish, the estimation of percentage of inter- and intra-specific SNPs was conducted based on the identification of SNPs from 1,000 randomly selected contigs. Of the 48,594 filtered SNPs identified from the all catfish assembly, over 18,000 (39%) were inter-specific SNPs; with 523 (1%) intra-specific SNPs for blue catfish, 2,352(5%) intra-specific SNPs for channel catfish, and 3,790 (8%) intra-specific SNPs for both channel catfish and blue catfish. However, approximately 17,000 SNPs could not be determined because overall the SNPs qualified as SNPs with at least two minor allele sequences, but only one of the minor allele sequences was from one of the two species of blue catfish or channel catfish (Table 7). Additionally, the number of inter-specific SNPs may be overestimated, due to failure to capture minor allele sequences from one or both species in the current EST data. However, the sequence differences between species should be greater than those within species. Although a large number of filtered inter-specific SNPs were identified (18,000 out of 48,000 total filtered SNPs), they were identified from a relatively small number of contigs. The 18,000 filtered inter-specific SNPs were identified from approximately 2,800 contigs, with an average of 6.6 SNPs per contig.

**Table 7:** Estimation of proportions of inter-specific and intra-specific SNPs from the set of filtered SNPs identified from the interspecific all catfish EST assembly

SNP type*	From 1,000 random contigs	Estimated from the all catfish assembly	Estimated % of total filtered SNP
Inter-specific SNP^1^	430	18,731	39
Intra-specific SNP, blue catfish^2^	12	523	1
Intra-specific SNP, channel catfish^3^	54	2,352	5
Intra-specific SNP, blue catfish and channel catfish^4^	87	3,790	8
Undetermined^5^	383	16,683	34
Subtotal	966	42,080	87
			
SNP from only blue catfish ESTs^6^	NA	1,118	2
SNP from only channel catfish ESTs^6^	NA	5,396	11
Subtotal	NA	6,514	13
Total SNP	NA	48,594	100

*SNPs were identified from contigs containing at least four sequences with at least two sequences from either channel catfish or blue catfish in the all catfish EST assembly: ^1^where there were no intra-specific blue catfish SNPs or intra-specific channel catfish SNPs, but the sequence differed between the two species at the inter-specific SNP position; ^2^where there were SNPs within blue catfish, but not within channel catfish; ^3^where there were SNPs within channel catfish, but not within blue catfish; ^4^where there were SNPs within both blue catfish and channel catfish; ^5^undetermined because overall the SNPs qualified as SNPs with at least two minor allele sequences, but only one of the minor allele sequences was from one of the two species of blue catfish or channel catfish; ^6^these SNPs were identified from ESTs that have been sequenced from only one of the two species, blue catfish or channel catfish, to date.

## Discussion

This project represents one of the major milestones in catfish research, and brings the catfish EST resources to almost a half million sequences in GenBank [21-26]. This EST resource will prove useful for gene discovery, molecular marker development, and genetic linkage and comparative mapping, and it will help facilitate whole genome sequencing and annotation. Parallel EST sequencing in two closely related species, *I. punctatus *and *I. furcatus*, may also provide the material basis for the analysis of genome duplication and genome evolution, providing the basis for establishment of orthologies through phylogenetics analysis.

The most important outcome of EST sequencing is gene discovery. This project allowed identification of 70,717 unique sequences in channel catfish and 54,815 unique sequences in blue catfish. We also conducted EST assembly using ESTs from both channel catfish and blue catfish. Assembly of all the catfish ESTs resulted in 111,578 unique sequences. Comparison of channel catfish and blue catfish coding regions in this study indicated that the two species share, on average, 95% sequence identity. Therefore, combining genes identified from both species should provide a more complete picture as to what fraction of the catfish transcriptome was captured to date. Such an approach was taken also because of practical considerations. Hybrid catfish produced by inter-specific hybridization of channel catfish × blue catfish is one of the best production lines of catfish used in aquaculture, and many believe that industry-wide application of this hybrid may have a revolutionary impact on the catfish industry. One of the major catfish breeding programs is based on introgression of beneficial genes from blue catfish into channel catfish breeds. Genetic linkage mapping has been conducted in both the intra-specific resource families involving only channel catfish [14] and the inter-specific resource families made from backcrosses of the channel catfish × blue catfish hybrids [12,13].

Given the close phylogenetic relationship of blue catfish and channel catfish, we expected that many of the contigs from the blue catfish and channel catfish EST assembly would merge together in an all catfish EST assembly. However, the all catfish EST assembly generated 45,306 contigs, a much greater number than the contigs generated in either the blue catfish (22,009) or channel catfish (28,941) EST assembly. There could be several reasons for this major increase in contig numbers with the all catfish EST assembly. First, some ESTs belonging to the contigs were only sequenced in blue catfish but not in channel catfish, and vice versa; second, singletons in either blue catfish or channel catfish were brought together to form new contigs; third, sequence variations or splicing differences between the two species may have led to the formation of a larger number of contigs under our assembly parameters; fourth, ESTs derived primarily from transcript untranslated regions of the two species may differ sufficiently to prevent placement in the same contig. Thorough analysis of the dataset has revealed that all four of these factors contributed to the high number of contigs in the all catfish assembly.

Analysis of the all catfish unique sequences showed that a large proportion of the catfish transcriptome has been captured. BLASTX searches identified 37% of total transcripts with significant hits, similar to levels reported in the salmon EST project [30]. The 111,578 unique catfish sequences had hits to 54% to 57% of the unique proteins of zebrafish, medaka, and green-spotted pufferfish using a cutoff value of E-10. This percentage appeared at first glance to be lower than our expectations. We therefore carried out best-hit searches using identical parameters as those used with catfish but comparing protein coverage of species with complete genome (transcriptome) sequences to serve as reference points. We found that the *Tetraodon *protein set had significant hits to only 61% of medaka proteins. By comparison, our catfish data set had significant hits to 56% and 57% of medaka and *Tetraodon *proteins, respectively. Similarly, zebrafish Refseq proteins were searched against the human Refseq protein database. In this case, zebrafish proteins had significant hits to 36% of total unique human proteins, compared to 33% in catfish-human alignments (Table 3). These reference numbers indicate both the high coverage of the catfish transcriptome obtained in this project, and the limitations of simple homology searches given the rapid divergence of many genes following speciation and the complexity of genome-wide and local gene duplication events within teleost species. Given that the identity of only 37% of the unique catfish sequences could be characterized by homology searches, the utility of the dataset should increase significantly with whole-genome sequencing of Ictalurid catfish and additional sequencing in closely related species within the order of Siluriformes. Interestingly, over 40,000 unique catfish sequences containing an ORF did not have a significant hit by homology searches. Further work will be needed to characterize whether low homology rates in these sequences is due to short read length, the rapid evolution of the encoded gene, or 'catfish-specific' gene duplication and divergence.

Large-scale EST sequences provide an enormous resource for molecular marker development. This project allowed identification of over 20,000 microsatellites within ESTs, of which 13,375 were located within unique ESTs and had sufficient flanking sequences for microsatellite primer design for genotyping (Table 6). Therefore, these microsatellites will be a major resource for genetic linkage and comparative mapping [12]. In addition, over 300,000 putative SNP sites were identified, of which over 48,000 were identified from contigs with at least four ESTs and the minor sequence was represented at least twice (Table 7). The 48,000 filtered SNPs should be highly useful for the development of a SNP panel for whole genome association studies [37].

The parameters of quality SNP assessment may not be applied to the very large contigs. The utilization of a minor allele frequency of six for all the contigs containing 30 sequences or more resulted in higher SNP frequency from these contigs, such as 13.4 SNPs per kilobase in the contigs with 100 to 500 sequences, and 15 SNPs per kilobase in the contigs with 500 sequences or more. Information regarding contigs over 500 sequences can be found in Additional data file 1. High SNP frequency within these large contigs may be caused by the accumulation of sequencing errors or alignment of transcripts from multi-copy loci, so SNPs from large contigs will be avoided in future SNP genotyping.

## Conclusions

In this project, generation and assembly of channel catfish and blue catfish ESTs allowed the identification of 45,306 contigs and 66,272 singletons, and a large majority of the catfish transcriptome was captured. Whole genome sequencing of channel catfish and blue catfish is currently underway, and the comparison between genome and transcriptome sequences will enable better understanding of the gene structure and organization. The analysis of the inter-specific ESTs resulted in the identification of 20,757 gene-associated microsatellites and over 300,000 putative SNPs, of which over 48,000 were filtered SNPs with the presence of the minor allele at least twice. These SNPs have been utilized to design the first generation high-density SNP chips using Illumina iSelect HD SNP genotyping panels for genome association studies. The inter- and intra-specific SNPs identified from the all catfish EST dataset assembly will greatly benefit catfish introgression breeding selection and whole genome association studies.

## Materials and methods

### cDNA library construction, EST sequencing and processing

The cDNA libraries were constructed by consortium investigators using various tissues, organs, and cell lines, including stomach, muscle, olfactory tissue, trunk kidney, head kidney, gill, intestine, spleen, skin, liver, pituitary, ovary and testis (Table 1). Total RNA was isolated from experimental tissues, reverse transcribed using an oligo-dT primer, directionally cloned into either the pSPORT-1 (Invitrogen Corp., Carlsbad, CA, USA) or pDNR-Lib (Clontech Laboratories Inc., Mountain View, CA, USA) plasmid vectors, and electroporated into competent *Escherichia coli*. One library (CBPN) underwent subtraction for highly expressed clones, ten libraries were normalized, and one library (CBCZ) was processed without normalization. Clone selection, arraying, and sequencing of all 12 libraries were performed at the US Dept. of Energy - Joint Genome Institute. Both ends of the insert were sequenced using Big Dye Terminator (V3.1) chemistry (Applied Biosystems, Foster City, CA, USA), and low quality sequences were trimmed. Contaminant sequences (*E. coli*, mitochondrial, cloning vector, rRNA, tRNA) were filtered.

### EST assembly

Three separate assemblies were performed: blue catfish ESTs, channel catfish ESTs, and all catfish ESTs. The new EST sequences and existing EST sequences from channel catfish and blue catfish were clustered and assembled using the Paracel Transcript Assembler, based on the CAP3 assembler [38]. Repeat sequences and poly (A) tails were masked and annotated. Prior to assembly, all EST sequences were compared to 'seed' sequences, which were existing catfish full-length or partial cDNA sequences in GenBank. New sequences sharing 80% similarity to seed sequences were clustered and assembled at 95% identity with at least a 50-bp overlap to generate seed-cluster contigs. The seed cluster assembly reduced the number of sequences for final assembly in order to minimize computational requirements. The remaining EST sequences were then clustered based on local similarity scores of pairwise comparisons with a minimum 88% similarity of at least 100 bp. Clusters containing only one sequence were denoted as singletons. The EST clusters were assembled into contiguous sequences (contigs) by multiple-sequence alignment with 95% identity of at least a 50-bp overlap, and a consensus sequence was generated for each cluster. Multiple contigs could be generated from each cluster, since EST clusters may not share enough similarity over their entire length to be assembled as a single contig. Multiple contigs could also be generated when ESTs in the cluster represented splice variant forms or paralogs. Single ESTs remaining in a cluster after the formation of contigs were designated as cluster singletons. The unique sequences for each assembly included the seed-cluster contigs, cluster contigs, cluster singletons, and singletons. All the sequence assemblies are available upon request to the corresponding author.

### ORF searching, gene identification and gene ontology annotation

All unique sequences obtained after the assembly were analyzed by ESTScan [39] to search for ORFs, which could be used to distinguish coding and non-coding sequences [39,40]. The putative protein sequences were also generated at the same time by ESTscan, which could be used to analyze splice variation, determine paralogs, and assess gene families. All unique sequences were compared against the *nr *and Uniprot databases using BLASTX (cutoff E-value of 1E-10) to obtain the putative identity. The NCBI Refseq protein and Ensemble databases (zebrafish, medaka, *Tetraodon*, human, mouse, and chicken) were also used to annotate the unique catfish genes.

### Full length cDNA identification

Putative full-length cDNAs were identified by comparison to full-length genes and start signals in Uniprot databases using TargetIdentifier [34,41] with a cutoff E-value of 1E-5. Once the start codon (ATG) and poly (A) tail were identified, the cDNA sequence was considered a full-length cDNA.

### Microsatellite and SNP marker identification

All the unique sequences were used to search for microsatellite makers using Msatfinder [42] with a repeat threshold of eight di-nucleotide repeats or five tri-, tetra- penta-, or hexa-nucleotide repeats. Clones containing 50-bp sequence on both sides of the microsatellite repeat were considered sufficient for primer design [43].

All three assemblies were used for SNP identification using autoSNP [44]. The parameters for minimum minor allele frequency for SNP detection varied with the number of sequences in the contig. A sequence variation was declared a SNP when: a mismatch was identified in contigs with four or fewer sequences; the minor allele sequence existed at least twice within contigs containing 5 to 6 sequences; the minor allele sequence existed at least three times within contigs containing 7 to 8 sequences; the minor allele sequence existed at least four times within contigs containing 9 to 12 sequences, or the minor allele sequence existed at least five times within contigs with 13 or more sequences. One thousand contigs containing filtered SNPs were randomly selected to inspect the inter- and intra-specific SNP calls.

## Abbreviations

BAC: bacterial artificial chromosome; EST: expressed sequence tag; ORF: open reading frame; SNP: single nucleotide polymorphism.

## Authors' contributions

SW conducted the bioinformatic analysis and was involved in writing the manuscript; EP was involved in data analysis and writing the manuscript; JA was involved in data analysis; GW, PL, HK, CM, BS, MW, HL, YJ, YL, FC, JL, WW, PX, BS, PB, JQ, ZS, BB, YW, QW, TT, SN, SL, LW, LK, SQ, EB, NM, and JT were involved in one or more processes of library construction, normalization, subtraction, or original research planning, writing the original grant proposal or writing the manuscript; EL, PR, SL, MW and DR were involved in EST sequencing at the Joint Genome Institute and management and storage of sequencing clones and datasets; JT, LL, and DV assisted the bioinformatic analysis; ZL directed the entire project and was involved in data analysis and writing the manuscript.

## Supplementary Material

Additional file 1 SNP information for contigs with 500 sequences or more. Click here for file
